# Development of Personalized Non-Invasive Ventilation Interfaces for Neonatal and Pediatric Application Using Additive Manufacturing

**DOI:** 10.3390/jpm12040604

**Published:** 2022-04-08

**Authors:** Marit Bockstedte, Alexander B. Xepapadeas, Sebastian Spintzyk, Christian F. Poets, Bernd Koos, Maite Aretxabaleta

**Affiliations:** 1 Department of Orthodontics, University Centre of Dentistry, Oral Medicine and Maxillofacial Surgery within the University Hospital Tübingen, Osianderstr. 2-8, 72076 Tübingen, Germany; alexander.xepapadeas@med.uni-tuebingen.de (A.B.X.); bernd.koos@med.uni-tuebingen.de (B.K.); maite.aretxabaleta-santos@med.uni-tuebingen.de (M.A.); 2 Section Medical Materials Science and Technology, University Hospital Tübingen, Osianderstr. 2-8, 72076 Tübingen, Germany; s.spintzyk@fh-kaernten.at; 3 ADMiRE Lab-Additive Manufacturing, Intelligent Robotics, Sensors and Engineering, School of Engineering and IT, Carinthia University of Applied Sciences, 9524 Villach, Austria; 4 Department of Neonatology, University Children’s Hospital, Calwerstr. 7, 72076 Tübingen, Germany; christian-f.poets@med.uni-tuebingen.de

**Keywords:** ventilation mask, craniofacial malformations, facial scanning, intraoral scanning, customized medical device, design and manufacturing workflow, rapid tooling

## Abstract

The objective of this study was to present a methodology and manufacturing workflow for non-invasive ventilation interfaces (NIV) for neonates and small infants. It aimed to procure a fast and feasible solution for personalized NIV produced in-house with the aim of improving fit and comfort for the patient. Three-dimensional scans were obtained by means of an intraoral (Trios 3) and a facial scanner (3dMd Flex System). Fusion 360 3D-modelling software was employed to automatize the design of the masks and their respective casting molds. These molds were additively manufactured by stereolithography (SLA) and fused filament fabrication (FFF) technologies. Silicone was poured into the molds to produce the medical device. In this way, patient individualized oronasal and nasal masks were produced. An automated design workflow and use of additive manufacturing enabled a fast and feasible procedure. Despite the cost for individualization likely being higher than for standard masks, a user-friendly workflow for in-house manufacturing of these medical appliances proved to have potential for improving NIV in neonates and infants, as well as increasing comfort.

## 1. Introduction

Through growing availability and affordability of computer-aided design and computer-aided manufacturing (CAD/CAM) technologies, it is now possible to meet the increasing demands of personalized medical devices at the point of care [1]. Examples range from patient-specific medical instruments [2] and orthoses [3] to personalized protective equipment (e.g., protective mask) [4,5]. The individualization of therapeutic devices has certain advantages, such as a more precise fit and built-in flexibility to adapt the product to the end-user [6,7]. Combined with additive manufacturing (AM), a fast, on-demand in-house production of highly complex parts is possible [8]. Individualization of an already existing product can be performed by reverse engineering (RE), where the product is redesigned based on the original device [9]. Medical RE (MRE) is additionally characterized by the inclusion of patient data, i.e., medical imaging data, in the process [10,11].

MRE comes into play if standardized product dimensions do not meet the required fitting standard. This is especially true for pediatrics, where the anatomy of the patient varies considerably, prohibiting development of a standard. This situation becomes particularly challenging in children with congenital anomalies, such as craniofacial malformations [12]. These newborns may require non-invasive ventilation (NIV), e.g., continuous positive airway pressure (CPAP), which is the most common reason for rehospitalization of newborns with acute respiratory disease [13]. NIV allows ventilation support while avoiding problems related to intubation or mechanical ventilation, such as barotrauma or volutrauma [14]. Of the options available, pediatric units preferentially use NIV as the initial respiratory support [15]. NIV interfaces for children are commercially available in different configurations (Table 1), e.g., as oronasal masks, nasal masks and prongs [16].

Due to the non-availability of oronasal masks in pediatric sizes [19], nasal masks are the interface used most commonly in pediatrics [17]. Compared to prongs, applying CPAP via facial masks significantly reduces the need for mechanical ventilation [23]. Nasal masks and prongs, however, can be susceptible to air leaks, caused by an inadequate fit [24]. Additionally, more than 70% of patients with craniofacial anomalies treated with NIV suffered from skin complications [25]. This is particularly true for neonates and toddlers with craniofacial malformations, where proper fitting and positioning of traditional masks is challenging [26]. Examples of these conditions include Robin sequence and Treacher–Collins syndrome, where patients are suffering from obstructive sleep apnea (OSA) and might require NIV in severe cases [27,28,29]. In Robin Sequence, approximately one-third of reported cases receive CPAP [30]. The main problems associated with discomfort and air leaks in off-the-shelf masks are caused by face morphology and size [31,32]. The lack of fitting oronasal or nasal masks and prongs for young patients could, therefore, be overcome by creating patient specific NIV interfaces, with the goal of improving CPAP effectiveness [26,33,34].

For adults, several approaches already exist to produce fitted oronasal [35,36] and nasal masks using CAD/CAM technology [37,38,39]. Personalized masks were found to be useful for reducing complications and discomfort associated with traditional masks, such as pressure ulcers [40]. Furthermore, a lower price-to-performance ratio was reported [39]. Additionally, for complex cases, such as those with dysmorphic facial features or craniofacial malformations, adequate anatomical positioning of the masks is crucial [41,42].

For pediatric patients, different attempts to produce nasal masks by conventional methods can be found in the literature. These are based on facial impressions, which are uncomfortable and invasive for the patient [43,44]. A less invasive approach includes acquiring the anatomical data using a facial scanner. Willox et al. showed that hand-held scanners are adequate for scanning faces for customizable NIV interfaces and emphasized the importance of these interfaces for children [45]. There are, first, attempts of employing CAD/CAM technologies and MRE for face mask manufacturing in children [26,46,47]. Morrison et al. constructed a personalized CPAP mask insert to ensure a better fit for a patient with Treacher–Collins syndrome and reported a significantly higher CPAP effectiveness [26]. Carroll et al. developed a nasal mask for a two-month-old boy [46]. Both groups applied a similar workflow: scanning the patient, designing the mask, constructing a mask casting mold and, finally, casting the mask with silicone [26,46]. The design procedure and the degree of automation could not be deduced from their studies. Despite these different attempts, however, a fast, feasible and easy to adapt workflow to manufacture NIV masks which could allow the in-house creation of the necessary medical device has not yet been published. The current study aims to test a methodology and to present a manufacturing workflow for nasal and oronasal masks for neonates and small infants. The objective is to provide a certain degree of design automation by means of MRE and CAD/CAM technologies, which aims to guarantee a fast and feasible mask fabrication. We applied different MRE technologies for daily use in the hospital and compared manufacturing times. Facilities could then produce patient-specific masks for newborns, in-house. A detailed design process and workflow of a personalized nasal and oronasal mask is presented and fitting is qualitatively compared to a commercially available solution.

## 2. Materials and Methods

### 2.1. Design of the Base Mask

**Figure 1:**
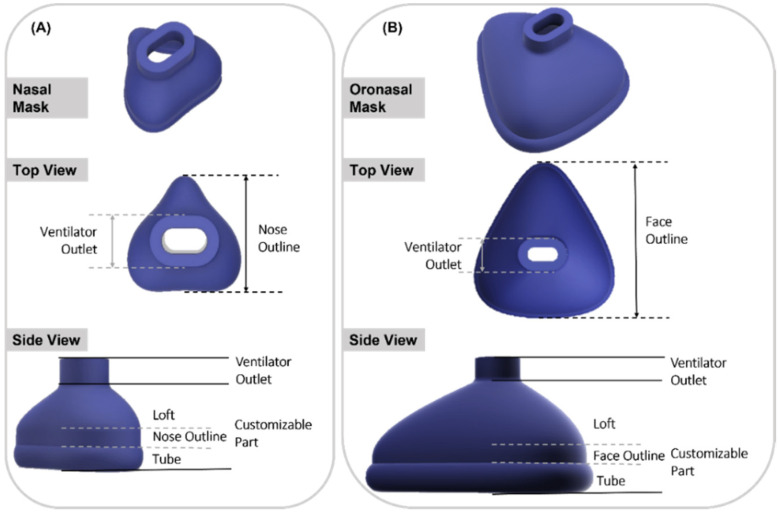
Isometric view of nasal and oronasal mask without face adjustment. (**A**) Nasal mask, top view and side view (from top to bottom); (**B**) Oronasal mask, top view and side view (from top to bottom).

To begin with, the base mask without any facial adaptation was designed in Fusion 360 CAD software (Autodesk Inc., San Rafael, CA, USA). The construction of both mask types consisted of two main parts: a standardized part containing the connection to the ventilator and a customized part. The connection to the ventilator was constructed to fit the standard CPAP system EasyFlow nCPAP (Fritz Stephan GmbH, Gackenbach, Germany). The customized part consisted of the nose outline (face outline in case of oronasal mask), loft and contact area. In the contact area of the mask, a short tube was designed to form the connection to the skin surface. The loft refers to the area between the connector and the nasal outline. Based on this design, base mask constructions were created for both nasal and oronasal masks, as shown in Figure 1.

### 2.2. Defining and Modifying Mask Parameters

**Figure 2:**
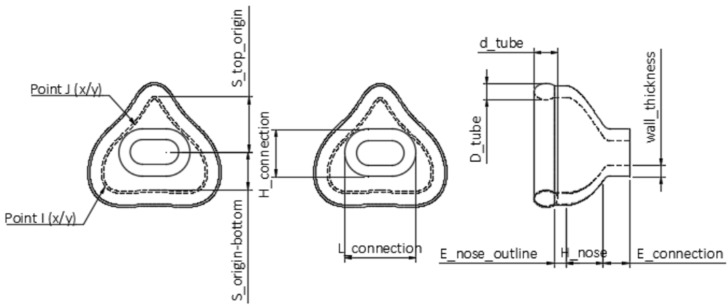
Technical drawing of the nasal mask obtained by Fusion 360. Top view in the left and middle images; side view in the right one. The scheme shows the user parameters.

Using the “Change Parameters” option, all relevant dimensions were saved as variables (called “User Parameters” in Fusion 360) that remain freely changeable within this separate window. In the sketches, the “Model Parameters” were connected to “User Parameters”, with the former being responsible for dimensioning bodies and sketches, while the latter were accountable for customizing parameters related to the individualization. These “User Parameters” were categorized as fixed and customizable parameters. Standard values, such as wall thickness, were classified as fixed parameters (Table 2). A standard or suggested value was given, e.g., 2.5 mm. A labeled technical drawing of the nasal mask can be seen in Figure 2. The oronasal mask was created from the nasal mask by increasing the customizable parameters.

### 2.3. User Workflow

**Figure 3:**
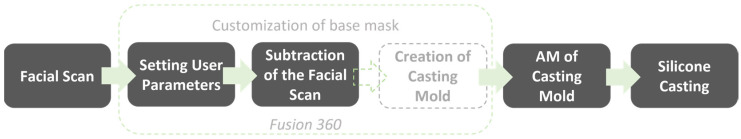
Final user workflow. Automatic steps are indicated by dotted lines.

The user workflow is displayed in Figure 3. It allowed to first choose between nasal and oronasal masks. The patient was scanned and the scan imported in CAD software Fusion 360, where it was trimmed and reduced. The next step was to customize the base mask. Mask parameters could be selected and the scan subtracted from the mask. After subtracting the scan, the mold was automatically created and then produced by AM and the mask by silicone casting.

### 2.4. Patient Data Acquisition and Mask Individualization

**Figure 4:**
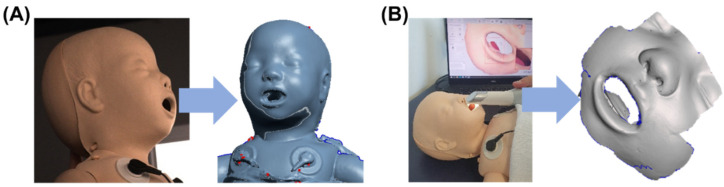
Facial scan of a neonatology dummy. (**A**) Process of scanning with the 3dMd facial scanner with corresponding stl. file; (**B**) Scanning with Trios3 intraoral scanner and respective stl. file.

To produce a prototype of the masks, a neonatal resuscitation training dummy (ALS Baby Trainer, Laerdal Medical GmbH, Puchheim, Germany) was scanned with two different technologies. A portable and a facial scanner were selected. The portable intraoral scanner (Trios 3, 3Shape A/S, Copenhagen, Denmark) was used to acquire data around the nose, while the facial scanner (3dMd Flex System, 3dmD Limited, London, UK) was employed for obtaining information on the complete face. The scan from the facial scanner was used for the oronasal mask, whereas that generated by the intraoral scanner was employed for the nasal mask. These two scanning processes are presented in Figure 4.

**Figure 5:**
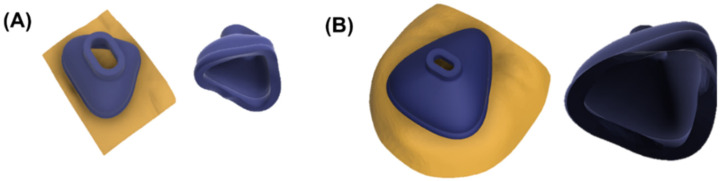
Fitted nasal and oronasal mask. (**A**) Nasal mask with (left) and without scan (right); (**B**) Oronasal mask with (left) and without scan (right).

Standard tessellation language (stl.) files were obtained from both scanners and imported into Fusion 360. The scan mesh was trimmed and reduced by the command “Reduce”. This step ensured maintaining maximum accuracy and reducing scan size so it would not exceed the computational limits of the software. Subsequently, the reduced scan was imported into the file containing the base mask design. The customizable parameters were adjusted and the mask was adapted to the respective scan by the command “Split Body”. Figure 5 shows the facial scan with the mask and the nasal (oronasal) mask after adjustment to the surface of the face.

### 2.5. Design and Manufacturing of the Casting Mold

**Figure 6:**
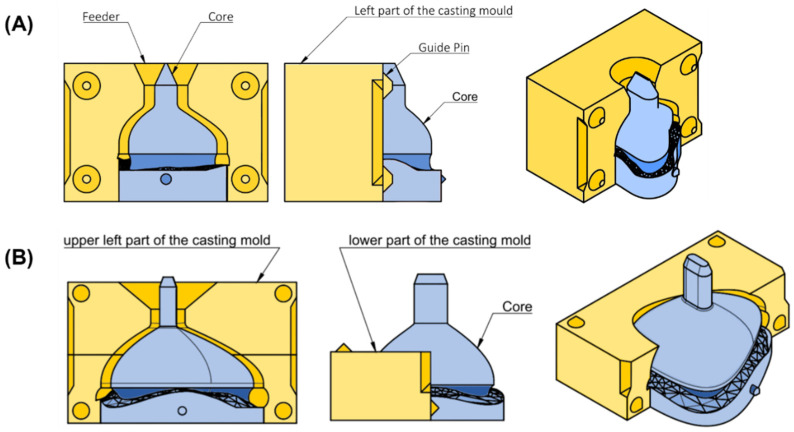
Technical drawing of casting mold for nasal and oronasal masks. (**A**) Isometric view (right), side view (middle) and front view (left) from casting mold for nasal mask; (**B**) Isometric view (right) with (left) and without (middle) upper left part from casting mold for oronasal mask.

To manufacture the mask from silicone, molds were created based on the individualized mask design. For both mask types, a casting mold was designed in Fusion 360. The dimensions of the mold depended on the mask parameters. Dimension included width (4.5 times the x-coordinate at point I), length (4 times S_top_origin) and height (sum of 2 times E_connection, 2 times H_nose and d_tube). When the user selected the parameters, the mold was automatically adjusted and the mask subtracted. The only major difference between the two mask molds was in the mask’s size. To enable pouring of silicone, molds were split into different sections. The casting mold for the nasal mask consisted of three parts, whereas the larger oronasal mask mold comprised five. The nasal mask mold consisted of two opposing parts and a core (Figure 6A). These three components were aligned using guide pins. For the casting mold of the oronasal mask, the left and right parts were additionally split into two halves. The mold also consisted of a core, as well as guide pins to ensure proper alignment for pouring (Figure 6B).

**Figure 7:**
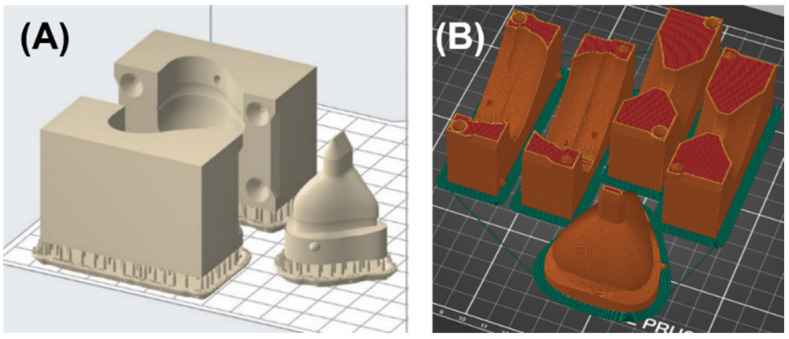
Orientation and position of the molding form on a building platform for nasal and oronasal mask. (**A**) Placement of molding parts as well as supporting structures for the nasal mask on the building platform in PreForm Version 3.12.2 (Formlabs, Sommerville, MA, USA); (**B**) Oronasal mask molding form parts on the building platform in PrusaSlicer Version 2.3.0 (Prusa Research a.s., Praha, Czech Republic).

Molds for nasal and oronasal masks were both produced with two AM technologies, stereolithography (SLA) and fused filament fabrication (FFF). The images below depict the mold for the nasal mask produced with SLA and the mold of the oronasal mask produced with FFF. Parameters, such as the guide pins’ tolerance and the taper angle, were modified in respect to each technology. The molds were manufactured employing the SLA device Form 3B (Formlabs, Sommerville, MA, USA) using the material Dental LT Clear Resin V1 (Formlabs, Sommerville, MA, USA. Lot #XG461ND1) at 0.1 mm layer thickness. The orientation and placement of the parts on the built platform are shown in Figure 7A. Support was automatically generated by the software. To manufacture the casting molds with this technology, a tolerance of 0.25 mm was chosen for the guide pins, while the feeder was designed with a taper angle of 25°. Additionally, the molds were produced using the FFF device i3 MK3S (Prusa Research a.s., Prague, Czech Republic). They were manufactured utilizing Polylactid Acid (PLA) filament (Prusament Vanilla White, Prusa Research a.s., Lot #52ad59fe76) at a layer thickness of 0.1 mm. The orientation of parts on the platform is shown in Figure 7B. No support structures were necessary. The tolerance for the guide pins was 0.1 mm and the taper angle 45°.

**Figure 8:**
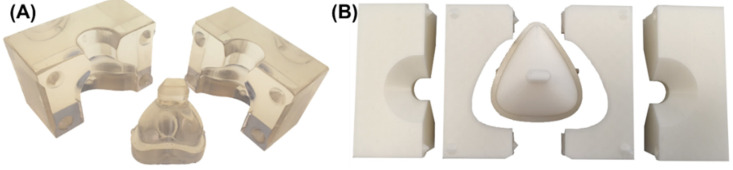
Manufactured molding form for nasal and oronasal mask. (**A**) SLA molding form for nasal mask (**B**) (from left to right) upper left part, lower left part, core, lower right, and upper right part of FFF molding form of oronasal mask.

SLA printed parts were post-processed (washing and light curing) following manufacturer’s instructions and support structures were removed after post-curing. The outer surface of the cast was smoothened using a grinding disk. Then, outer and inner sides of the parts were mechanically polished with muslin buff using powdered pumice and finished with polishing paste. The finished casting mold is presented in Figure 8A. The FFF mold parts were detached from the printing platform and the brim was removed. No further post-processing was necessary here as the fit was sufficient (Figure 8B).

### 2.6. Manufacturing of the Silicone Mask

**Figure 9:**
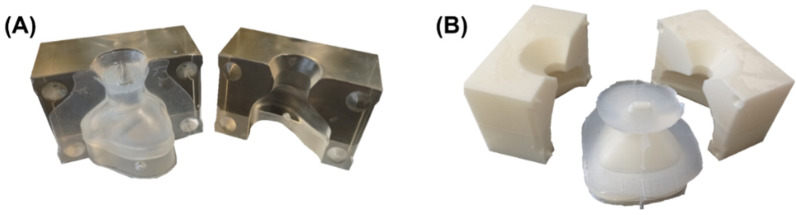
Casting mold for nasal and oronasal mask with cured silicone. (**A**) Nasal mask; (**B**) Oronasal mask.

An addition-curing, two-component 45 ShoreA silicone (SF45, Silikonfabrik, Ahrensburg, Germany. Lot #200121 and #290121) was poured into the molds. Trapped air bubbles were removed using a vibrating plate and vacuum pot. To prevent the form from slipping or rising, it was tightened with a rubber band. To fill the FFF mold for the oronasal mask, two steps were required. First, the core was connected to the two lower parts of the mold, silicone inserted and bubbles removed as described above. Then, silicone was injected into the second part and air bubbles were removed as well. The silicone mold was cured in an oven at 50 °C for 15 min. Once the silicone was fully cured, the mold was opened, and the finished mask prototype removed (Figure 9). Silicone overflow was trimmed using a scalpel.

The required time of all processing steps was recorded during the manufacturing of one nasal and one oronasal mask. Moreover, the fitting of the mask was evaluated by placing the final masks on the face of the resuscitation dummy, directly compared to the fit of the conventionally produced mask (RD806-10, Fisher & Paykel Healthcare Limited, Auckland, New Zealand). Furthermore, it was checked whether connecting the mask to the standard applicator is possible.

## 3. Results

### 3.1. Required Time

The time required to personalize the nasal and oronasal mask is detailed in Table 3. In addition, manufacturing time for the nasal and oronasal mask with both AM technologies (SLA or FFF) is listed. The hands-on time comprises scan preparation, mask and casting mold design, mold post-processing, silicone casting and finally, mask post-processing. Hereby, the total hands-on time for the nasal mask produced with SLA was approximately 1.5 h (0.5 h with FFF), while the complete workflow (from receiving the scan to the finished mask) was approximately 6 h (9 h with FFF). In case of the oronasal mask produced with SLA, the hands-on time was 1.75 h (1 h with FFF), whereas more than 8 h were required for completion (more than 16 h with FFF). Less time was required for postprocessing the FFF molds, resulting in a shorter hands-on time. The duration of casting process and finishing of the masks was independent of the employed AM technology for mold manufacturing. With FFF, printing required considerably more time which was not compensated by the shorter hands-on time.

### 3.2. Mask Fit

**Figure 10:**
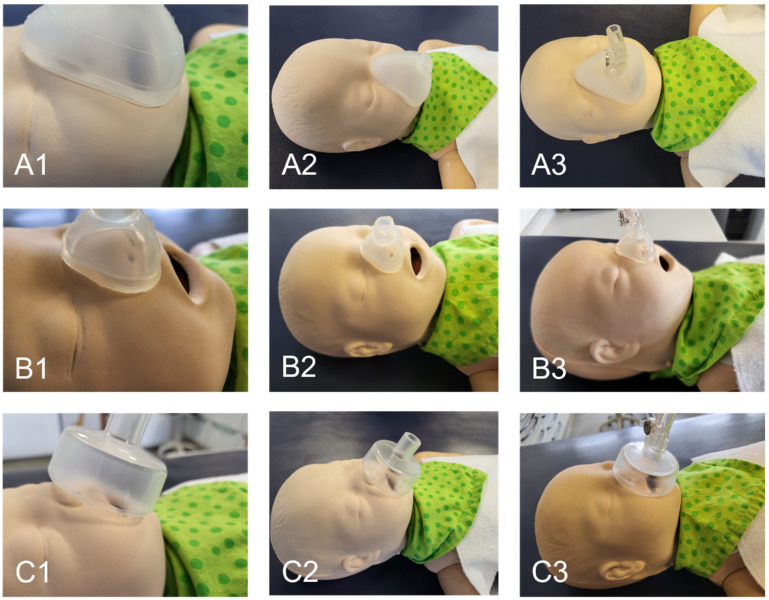
Fitting of mask to the neonatal dummy. (**A**) Oronasal mask; (**B**) Nasal mask; (**C**) Conventional oronasal mask.

An image of the nasal mask on the dummy’s face is shown in Figure 10. It is evident that the manufactured nasal and oronasal masks (Figure 10A,B) have an improved adaptation to the skin surface compared to the conventional mask (Figure 10C). The applicator of the EasyFlow nCPAP system could easily be connected to all three masks (Figure 10(A3,B3,C3)). The mask created by the SLA mold was clearer than that produced with the FFF mold.

## 4. Discussion

In this article, a method is presented to individualize nasal and oronasal masks for ventilating neonates and infants as an alternative to using conventional, non-customized masks, including the entire process from receiving a patient’s facial scan, designing the individualized mask, as well as manufacturing and, finally, evaluating its fit.

### 4.1. Comparison of the Proposed Design to Other Customizable Masks

There have been numerous attempts at defining workflows for designing and manufacturing masks for children and adults. Despite this, no comparable study providing a feasible and affordable in-house workflow for neonatal application could be found. In the following section, the presented workflow is compared to other potential options.

Concerning nasal masks, two methods have been proposed. Cheng et al. created a nasal cushion for CPAP in adults using CAD, from which, later, a mold was manufactured by Polyjet AM and shape deposition manufacturing. They obtained the facial dimensions by digitalizing a conventional face impression [39]. In another study, they applied CNC techniques to produce the respective mold [37]. Their findings suggested that a better fit can be obtained with the customized cushion compared to commercially available products [37,39]. Despite proposing a feasible workflow, performing a facial impression on neonates and infants is a rather invasive procedure in comparison to facial scanners. Moreover, CAM technologies, such as Polyjet AM, are not as affordable and accessible for hospitals as desktop AM devices, such as the SLA technology used in the presented workflow. In addition, a larger variety of medically certified materials are commercially available for SLA-based printers. Operating these devices has been described as user-friendly [48]. Milling machines are widely used in hospitals in fields such as dentistry. These milling devices are normally intended for manufacturing parts of dimensions much smaller than the presented mold and, therefore, would not suffice. Consequently, an industrial CNC milling machine might be necessary which would require outsourcing production, which results in a longer duration and additional costs. Generally, the shape of the mold might be too complex to be milled where AM has much less dimensional restrictions. Carrol et al. reported the customization of a nasal mask for a two-month-old child with Down syndrome. After modifying a 3D-scanned conventional mask to fit the patient’s face, a mold was created and manufactured using AM. Unfortunately, no further data, such as the used printer, could be obtained from their study [46]. This proposed design, however, does not allow modification of the mask based on some predefined options and parameters. This could make the workflow more difficult to implement than the current workflow. In addition, it has limits concerning scalability and standardization.

For oronasal masks, two other methodological reports were found in the literature. Both are based on the principle of modifying commercially available masks by implementing a new interface between face and mask. Morrison et al. created a NIV interface for a single patient with Treacher–Collins syndrome. They designed an individualized inlay for a generic mask (printed with FFF), with the objective of reducing leakage and improving fit [26]. Despite proposing a workflow solution for neonates or infants with craniofacial anomalies, the design procedure cannot be easily modified. Morrison et al. implemented a procedure where the complete design of the inlay had to be created starting from the scanned surface. A more feasible solution is only modifying selected parts of the mask for individualization, such as modifying the parameters as in the proposed workflow. Wu et al. performed a single case study where an oronasal mask for patients with variant amyotrophic lateral sclerosis was created. Their construction consisted of a generic mask’s hard shell, a silicone layer and an interface between hard shell and silicone layer [35]. Modifying a generic mask is unfortunately not suitable for preterm infants, who are the beneficiaries of the presented workflow, as there are only few commercial oronasal masks available [19].

### 4.2. Potential of the Workflow

The proposed design and manufacturing workflow may be a viable solution for in-house manufacturing of personalized masks. Based on facial scans from the patient, an individualized medical appliance could be fabricated. Two data acquisition devices were considered: an intraoral scanner and a facial scanner. The intraoral scanner is a more affordable solution, which might be available in every hospital with a dental department nowadays. Moreover, these scanners are mostly portable and could even be used in an intensive care setting. The intraoral scanner poses an advantage for scenarios where the patient cannot be move from the ventilation device, other devices, or treatments. However, high-quality scans can be challenging in babies that are constantly moving, particularly for oronasal masks. Especially when crying, the region around the mouth is in constant movement. In some cases, these devices might still be sufficient for acquiring a scan of the nasal mask region.

With state-of-the-art technology, facial scanners might be a better choice, despite some disadvantages. Although facial scanners are more expensive, they allow for recording a video of a moving subject and enable to then obtain a 3D image from a single video frame. Therefore, especially in moving patients the best 3D image can be selected after the scan procedure. Other options range from recording the face with a smartphone camera to more sophisticated handheld 3D Scanners. It thus remains a case-by-case decision which scanning method is preferred.

Independent of the method used for data acquisition, the workflow can be applied to any facial 3D model.

The software employed for the design procedure (Fusion 360) is a widely accessible CAD program that is currently free for home or educational users [49]. This allows for the developed method to be made more readily available to hospitals. Additionally, the design of the mask can be adapted as well to allow addition of mask fixing systems, avoid areas, such as feeding tubes, applying different shore hardness of silicone, adapting mask wall thickness, etc.

Referring to the manufacturing procedure, access to an AM device for mold production is necessary. In this study, two widely accessible devices were employed: SLA and FFF. Although SLA can provide molds with higher accuracy and surface finishing than FFF, it is considerably more expensive and requires a more timely post-processing [48]. The mold manufactured by a SLA-based printer also required manual post-processing, hence longer hands-on time and more additional equipment is required. In this study, a completely transparent silicone nasal mask could only be obtained by employing the SLA technology, whereas an opaquer oronasal mask was obtained by using the FFF manufactured mold. This could be accounted to the rougher surface of the FFF mold. Another advantage of molds manufactured by SLA devices is that the mold is transparent, which facilitates locating trapped air bubbles. Currently, a wider range of medically approved materials are commercially available for SLA in comparison to FFF. Despite of this, with increasing improvement of devices and materials for FFF, this situation is expected to change within the next few years. Moreover, direct printing of the mask without a mold could be a valid alternative. Some methods for direct printing of silicone have been utilized for different applications (silicone, drop on demand, etc.). Medical-grade silicone printers could also be employed for direct printing of the mask [50]. They are still expensive, and therefore, the objective of creating a feasible but fast workflow would not be possible [51]. Direct printing of masks with SLA technology is still in the future as there are no flexible, medically approved materials on the market. With further development of materials and devices in the following years, direct printing of masks by AM technologies might be possible [52].

In the first instance, this study aimed to provide a scalable workflow for individualized masks for newborns and small infants, as well as patients with craniofacial anomalies. Even though, the proposed design method can also be used to create customized masks for patients of all sizes. Despite that standardized masks are more readily available for adult patients, prolonged ventilation can lead to skin irritation and creation of pressure ulcers [53]. Customized masks are known to decrease leakage and may improve the fitting as well as comfort [26,53].

Alongside with the advantages of this workflow, some disadvantages must be mentioned. For example, individualization of any type of medical product is often closely related to increased cost. Even though studies show a higher price-to-performance ratio, not all facilities might have the required financial and material resources [37]. Even though, for facilities without access to a printer, the production or the casting mold could be outsourced. The workflow was presented in a way that users with no previous CAD-knowledge could work with it and the presented devices are user-friendly. However, dealing with digital technologies does require an additional learning stage for the facility’s staff [54]. Regarding the workflow, it was automatized as much as possible, but still manual processing steps are required, such as: post-processing of the molds, pouring of the silicone, and the removal of the seam created by casting in the silicone mask.

### 4.3. Limitations

It is important to recognize some limitations of the study. First, the employed silicone was not a medically approved material. Due to the increased price for the approved one, a normal commercial silicone was used to test the presented methodology. In addition, because of ethical implications of working with such patients, a resuscitation dummy was employed as a patient to assess the feasibility of this workflow. Therefore, the scanning and digital design was carried out on a non-moving subject. Thus, the use of these masks for NIV, its leakage and fitting in real patients remains to be tested following the respective ISO standards [55].

## 5. Conclusions

The current study describes a feasible design and manufacturing workflow for patient-specific oronasal and nasal masks for use in NIV. The presented solution was fast and easy enough, so that users without previous CAD knowledge would be able to individualize the designs to the patients. Moreover, the manufacturing could be carried out in-house to give a solution for neonates and infants, as well as patients with dysmorphic features or craniofacial anomalies, where the sizing and shape of standard masks is not suitable. The design workflow was proposed so that a standardized pre-designed mask could be fitted to every patient, by using an acquired scan from the patient and a changeable user parameter interface. Despite the fact that costs for individualization are expected to be higher compared to the standard masks, this is outweighed by the potential benefit for the patient where a user-friendly workflow for in-house manufacturing of these medical appliances has a lot of potential.

## 6. Outlook

Although this study concludes with a simple and feasible workflow for mask creation and manufacturing, further studies are needed to achieve its implementation into daily clinical practice. For a start, the mask must be validated regarding international standards. The workflow must also be employed in a real patient scenario, where leakage and fitting is studied.

This current workflow aims to keep costs to a minimum and use resources that could be available in most facilities without the need for outsourcing. Nonetheless, scanning and AM technologies are rapidly improving. Therefore, the workflow could be optimized as the technology evolves. Concerning scanning, the use of smaller portable devices or smartphone 3D cameras is soon to be conceivable as the basis of the workflow. Regarding the designing of the mask, the program could be adapted by implementation of tools, such as user interfaces, so that the design experience would be more user friendly and possibly even faster. Referring to the manufacturing, a two-step manufacturing process was used in this study, where the mask was not directly produced, and, instead, the fabrication of a mold and casting with silicone were necessary. The main reason was to have an affordable process that could be carried in-house in almost all facilities. However, as flexible materials become more common for AM or as soon as silicone printers are more affordable, the direct manufacturing of the mask and the avoidance of the mold would be possible.

## Figures and Tables

**Table 1:** Interfaces for non-invasive ventilation in pediatrics with advantages and disadvantages [14,17,18,19,20,21,22].

NIV Interface	Nasal Mask	Oronasal Mask	Prong
	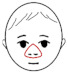	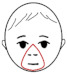	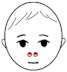
Advantages	Allows communication Newborn sizes available	No mouth leak	Allows communication Neonatal sizes available
Disadvantages	Risk of mouth leak Risk of skin injury	Few newborn sizes available Risk of skin injury	Painful procedure Risk of mouth leak Nasal injuries Difficult to secure position More traumatic

**Table 2:** Summary of the customizable and fixed parameters for nasal masks.

Fixed Parameter	Abbreviation	Standard Value
Height of connection	H_connection	12 mm
Length of connection	L_connection	18 mm
Wall thickness	wall_thickness	2.5 mm
Height of extrusion of connection	E_connection	6 mm
**Customizable parameter**	**Abbreviation**	
Height of the nose	H_nose	
Space between the top of the nose outline and the origin	S_top_origin	
Space between the bottom of the nose outline and the origin (0/0/0)	S_bottom_origin	
x-coordinate of point I	I_X	
y-coordinate of point I	I_Y	
x-coordinate of point J	J_Y	
coordinate of point J	J_X	
Height of extrusion of nose outline	E_nose_outline	
Width of the tube	D_tube	
Height of the tube	d_tube	

**Table 3:** The required time for manufacturing a single nasal and oronasal mask. The time was taken for FFF and SLA-method. Hands-on time consists of the steps indicated by *.

Workflow		Time Required		
	Nasal Mask		Oronasal Mask	
	SLA	FFF	SLA	FFF
Preparatory scan *	10 min		12 min	
Creating mask and casting mold *	8 min		10 min	
AM of casting mold	4 h 15 min	8 h 12 min	6 h 45 min	15 h 38 min
Postprocessing of the mold *	1 h	1 min	1 h	1 min
Casting the silicone *	10 min		2 × 10 min	
Curing the silicone	15 min		15 min	
Postprocessing the nasal mask *	4 min		4 min	
In total * Hands-on time	6 h 2 min 1 h 32 min	9 h 33 min	8 h 46 min 1 h 46 min	16 h 40 min 47 min

## Data Availability

The data that support the findings of this study are available from the corresponding author, Marit Bockstedte, upon reasonable request.
